# Comparison of Soft and Hard Tissue Outcomes Between Root‐Analog Zirconia and Conventional Zirconia Implants: A Prospective Clinical Study

**DOI:** 10.1155/ijod/5104539

**Published:** 2026-05-23

**Authors:** Ammar Almarghlani, Amirah Aldosari, Abdullah Alfarran, Rayan Sharka, Hassan Abed, Badr Othman, Ali Alghamdi

**Affiliations:** ^1^ Department of Periodontics, Faculty of Dentistry, King Abdul-Aziz University, Jeddah, Saudi Arabia, kau.edu.sa; ^2^ Department of Oral and Maxillofacial Surgery, Faculty of Dental Medicine, Umm Al-Qura University, Makkah, Saudi Arabia, uqu.edu.sa; ^3^ Department of Basic and Clinical Oral Science, Faculty of Dental Medicine, Umm Al-Qura University, Makkah, Saudi Arabia, uqu.edu.sa

## Abstract

**Objective:**

To compare oral hygiene parameters, crestal bone stability, and early postoperative outcomes between zirconia root‐analog implants (RAIs) and conventional zirconia implants (CZIs) over 3 months.

**Materials and Methods:**

This prospective, controlled clinical study included 30 participants assigned to either an RAI or CZI group based on anatomical suitability for fabricating a custom RAI. RAIs were designed from digital scans of the extracted roots and milled from zirconia, while the CZI group received standardized Straumann PURE Ceramic Implants. Clinical indices and cone‐beam computed tomography (CBCT)‐based radiographic measurements were recorded at 1 and 3 months. Data were analyzed using independent *t*‐tests and chi‐square tests with a significance level of *p* < 0.05.

**Results:**

No significant differences were observed in plaque index (PI) or bleeding on probing (BOP) between groups at 1 and 3 months. Crestal bone height remained stable for both implant types, with only a slight reduction in CZIs. RAIs demonstrated reduced postoperative swelling, pain, and inflammation at 1 month. Soft tissue indices and bone levels were comparable across both groups at 3 months.

**Conclusion:**

RAIs offered improved early patient comfort and bone preservation, suggesting a potential trade‐off between mechanical reliability and biological performance. Further long‐term studies are needed to optimize RAI design and clinical protocols.

## 1. Introduction

Conventional zirconia implants (CZIs) have become increasingly favored over titanium implants due to their advantageous biological and esthetic properties [1, 2]. These include high biocompatibility, a tooth‐like coloration that supports a natural appearance, and strong corrosion resistance [3, 4]. Such characteristics make zirconia particularly suitable for patients with elevated esthetic expectations, especially in the anterior region [5]. The material’s inert nature also contributes to favorable tissue responses, reducing the likelihood of inflammatory reactions [6, 7]. Beyond esthetics, zirconia implants have shown promising clinical outcomes in bone preservation, implant stability, and peri‐implant soft tissue health [2, 8]. Evidence from clinical studies supports their ability to achieve reliable osseointegration, critical for long‐term implant success [1, 2, 8, 9]. Radiographic evaluations further suggest that zirconia implants help maintain the surrounding bone volume and minimize peri‐implant bone resorption, contributing to their overall stability and longevity [10–12].

Although CZIs have demonstrated reliable clinical and esthetic outcomes, emerging implant designs, such as root‐analog implants (RAIs), aim to address limitations in anatomical conformity and surgical invasiveness [7, 13]. RAIs are custom‐fabricated to replicate the morphology of the extracted tooth root, allowing for a more anatomically congruent fit within the alveolar socket [14]. This anatomical congruence minimizes bone resorption and soft tissue regression, promotes superior osseointegration, and reduces surgical trauma through precise placement [13, 15, 16]. Additionally, RAIs facilitate optimal stress distribution, contributing to reduced peri‐implant bone loss and improved biomechanical stability [17, 18]. One‐piece RAIs promote healing, reduce biofilm formation, soft tissue inflammation, and the likelihood of recession, and achieve osseointegration comparable to that of titanium implants; however, long‐term predictability remains under investigation [13, 19–21].

A major advantage of RAIs is their lack of reliance on bone drilling [6, 22]. This significantly reduces trauma, enhances postoperative recovery, and promotes faster healing [6, 22]. By fitting precisely into the extraction socket, RAIs reduce the need for procedures such as bone augmentation, which are commonly required in conventional implantology [15, 16, 21, 22]. Preserving natural bone architecture enhances osseointegration and reduces postoperative discomfort [13, 22]. Moreover, atraumatic placement contributes to faster healing and lowers the risk of postoperative complications, such as inflammation and swelling [23]. The absence of drilling minimizes soft tissue disruption, increases peri‐implant stability, and reduces the need for interventions such as sinus lifting or bone grafting [16]. These benefits not only reduce surgical time and cost but also improve patient comfort [24]. Finite element analyses of contemporary RAIs designs, including fin‐ and bulb‐shaped press‐fit geometries, further demonstrate favorable stress distribution and enhanced primary stability in both maxillary and mandibular posterior regions [25]. RAIs also offer significant advantages in soft tissue. Their close fit within the socket minimizes dead space, thereby reducing the risk of biofilm accumulation and peri‐implantitis [15, 21]. Moreover, the smooth zirconia surface promotes soft tissue attachment, enhances mucosal stability, and reduces soft tissue recession [18]. RAIs also preserve both hard and soft tissues, contributing to long‐term success and reducing the need for secondary procedures [7]. Additionally, validated three‐dimensional finite element models show that multirooted RAIs generate lower stress and reduced microstrain and exhibit superior primary and secondary stability compared with conventional threaded implants [26].

Despite the documented advantages of RAIs, their clinical application remains challenged by the absence of standardized protocols for fabrication and placement. Variability in implant design, material choice (e.g., titanium versus zirconia), and surface modification techniques (e.g., sandblasting or macroretentive features) can contribute to inconsistent clinical outcomes [27]. Although technologies like cone‐beam computed tomography (CBCT) and computer‐aided design/computer‐aided manufacturing (CAD/CAM) have enhanced procedural accuracy, the lack of unified guidelines continues to affect the predictability and reproducibility of zirconia RAI placement [27]. While some studies have reported favorable success rates, others have documented early failures, underscoring the need for further investigation and protocol refinement [22]. Moreover, although zirconia RAIs have shown encouraging results, there remains a significant gap in the literature regarding their comparative effectiveness relative to CZIs. This study aims to address this gap by evaluating whether zirconia RAIs offer superior clinical outcomes across multiple soft‐ and hard tissue parameters. The research questions posed in this study were as follows:

Do zirconia RAIs demonstrate comparable stability and improved postoperative outcomes compared to CZIs?

The null hypotheses of the study were as follows:

H_01_: There is no significant difference in the stability of zirconia RAIs compared to CZIs.
H_02_: There is no significant difference in postoperative complications between zirconia RAIs and CZIs.

H_01_: There is no significant difference in the stability of zirconia RAIs compared to CZIs.

## 2. Materials and Methods

### 2.1. Study Design and Setting

This prospective, comparative clinical study was designed to compare zirconia RAIs with CZIs. All participants were recruited consecutively from patients presenting with a single tooth requiring extraction and immediate implant rehabilitation. A standardized clinical and radiographic assessment was used to confirm eligibility.

Participants were allocated to one of the two groups based on the clinical feasibility of fabricating an RAI. Individuals with a tooth exhibiting normal root morphology suitable for digital scanning were assigned to the RAI group, while those with an unsuitable anatomy were allocated to the CZI group. As the allocation depended on anatomical suitability, the study did not involve randomization, and allocation concealment was not applicable.

All study procedures adhered to the Declaration of Helsinki and were approved by the Ethics Committee of King Abdulaziz University (Approval Number: 172‐11‐24). All participants provided written informed consent. The investigation was conducted at a private dental clinic in Jeddah, Saudi Arabia, from December 2024 to September 2025. This study was reported in accordance with the Strengthening the Reporting of Observational Studies in Epidemiology (STROBE) guidelines, and the completed STROBE checklist is provided as Supporting File.

### 2.2. Study Participants

All patients were screened using a standardized clinical and radiographic assessment. The inclusion criteria were as follows:

• Required the replacement of a single missing tooth.
• The tooth to be replaced was single‐rooted (anterior or premolar region).
• Adequate bone at the implant site confirmed by CBCT (sufficient height and width for placement without grafting).
• Bone quality corresponding to Lekholm and Zarb types I–III [28].
• Systemically healthy.
• Nonsmokers.
• No medical conditions affecting bone healing or soft tissue response.
• For the RAI group, the extracted tooth had to have a normal root morphology suitable for digital scanning and milling.

The exclusion criteria included the following:

• Active oral infection at the surgical site.
• Untreated periodontal disease.
• The history of radiotherapy to the head and neck region.
• Parafunctional habits (e.g., bruxism).
• Pregnancy or lactation.
• Any contraindications to oral surgical procedures or implant placement.

The required sample size was calculated using 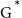
Power (Version 3.1). Effect size estimates were derived from previous clinical studies comparing implant or abutment groups with similar outcome measures, including soft tissue indices, esthetic scores, and bone‐level changes [29, 30]. Studies with comparable two‐group clinical designs have reported medium effect sizes (Cohen’s *d* ≈ 0.60–0.80) for parameters such as PES scores and peri‐implant soft tissue outcomes in groups of 14–16 participants per arm. Based on an anticipated effect size of *d* = 0.75, with *α* = 0.05 and power = 80%, the minimum required sample size for the present study was 15 participants per group (total *n* = 30).

### 2.3. Preoperative Assessment

All participants underwent a comprehensive clinical and radiographic evaluation before implant placement. CBCT imaging was performed preoperatively using a standardized protocol with a voxel size of 0.2 mm. Bone height measurements were taken using consistent anatomical landmarks, defined as the linear distance from the crestal bone level (the most coronal point of the alveolar crest) to the apical end of the extraction socket along the long axis of the planned implant site. Measurements were obtained in the sagittal plane corresponding to the tooth’s native root trajectory to ensure reproducibility.

All radiographic measurements were performed using the Romexis Viewer software (Planmeca, Helsinki, Finland) with its calibrated digital ruler automatically scaled to millimeters. The measurement grid and magnification tools were used to ensure accuracy and standardization across participants.

To ensure consistency of radiographic analysis, all measurements were performed by a single examiner with prior experience in CBCT interpretation. Before data collection, the examiner completed a calibration and standardization session, during which 10 representative CBCT scans were measured twice and compared for consistency. Intraobserver reliability was evaluated by reassessing 20% of all CBCT scans 2 weeks apart. The intraclass correlation coefficient (ICC) demonstrated excellent reliability (ICC = 0.89), indicating strong reproducibility of the bone height measurements in this study.

Oral hygiene status was evaluated by measuring bleeding on probing (BOP) and the plaque index (PI) to ensure suitability for implant placement and establish a baseline for subsequent follow‐ups.

### 2.4. Intervention Protocols

#### 2.4.1. Experimental Group

**Figure 1:**
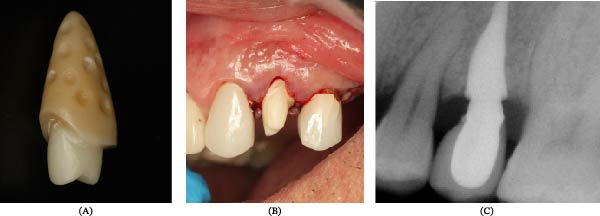
(A) Three‐dimensional CAD/CAM rendering of the milled root‐analog implant (RAI), generated from a high‐resolution intraoral scan of the extracted root; digital measurements were based on a millimeter‐calibrated design environment. (B) Clinical photographic view showing the insertion of the RAI following atraumatic extraction; soft tissue landmarks included for spatial reference. (C) Periapical radiograph obtained using a standardized long‐cone paralleling technique; radiographic scale calibrated in millimeters for assessment of implant position and crestal bone alignment.

The extracted tooth root was scanned using a high‐resolution chairside intraoral scanner (Medit i700, 5–10 μm accuracy) to obtain an accurate digital representation of its morphology. The data were exported in STL format and imported into CAD software (Exocad DentalCAD, Exocad GmbH, Germany) for the fabrication of the customized RAI. The RAI was designed to replicate the native root anatomy while incorporating controlled dimensional adjustments. A uniform circumferential enlargement of 0.5 mm was applied to compensate for the periodontal ligament space, except on the buccal aspect, where no enlargement was introduced to preserve the buccal bone plate (Figure 1A). The digital design also incorporated macroretentive concavities, ~1.0–1.5 mm in diameter and 0.5–0.8 mm in depth, strategically positioned along the middle and apical thirds to enhance mechanical interlocking after insertion.

The finalized digital design was then transferred to a five‐axis zirconia milling system (imes‐icore CORiTEC 250i, Germany) for milling from medical‐grade, fully sintered zirconia discs (1400 MPa flexural strength). The implants were sandblasted with 50 µm aluminum oxide at 1.5–2.0 bar, producing a microroughened surface with an estimated Ra of 1.5–2.0 µm, suitable for promoting early osseointegration.

After final sintering in accordance with the manufacturer’s temperature protocol, each implant was inspected for dimensional accuracy, surface uniformity, and the integrity of macroretentive features before undergoing disinfection and sterilization.

##### 2.4.1.1. Initial Cleaning

The implants were manually cleaned with a soft, sterile brush under sterile water to remove any residual debris, then rinsed with sterile saline or sterile distilled water to eliminate any remaining particulates.

##### 2.4.1.2. Chemical Disinfection

The implants were chemically disinfected by first immersing in 70% ethanol for 10 min, followed by immersion in 3% hydrogen peroxide for 5–10 min. Hydrogen peroxide‐based disinfection preserves the surface integrity of the implant and maintains the biological compatibility of zirconia [31].

##### 2.4.1.3. Thermal Sterilization

The implants were subjected to dry heat sterilization at 180°C for 1 h to ensure complete sterilization, including the inactivation of bacterial spores and heat‐resistant organisms. Zirconia surfaces exhibit no significant structural or morphological changes following combined chemical disinfection and heat sterilization [32–35].

##### 2.4.1.4. Final Rinsing and Storage

The implants were rinsed three times with sterile saline before storage in sterile saline or 0.02% chlorhexidine solution until the time of surgical placement, to maintain sterility and prevent biofilm formation.

The implant was inserted 24 h postextraction. During this time, the patients were instructed to avoid manipulating the extraction socket and to maintain cleanliness by gently rinsing with sterile saline. They were also advised to chew on the contralateral side. Bleeding was induced from the apical area before insertion. The custom implant was inserted by tapping to achieve a snug fit (Figure 1B). Periapical (PA) radiographs were taken to confirm appropriate implant positioning (Figure 1C). The patients were instructed not to rinse on the day of surgery; gentle toothbrushing at the surgical site using the Modified Stillman Brushing Technique was initiated the following day, followed by twice‐daily brushing thereafter. Patients were also advised to avoid hard foods and to perform gentle saline rinses after meals beginning on the second postoperative day.

#### 2.4.2. Control Group

**Figure 2:**
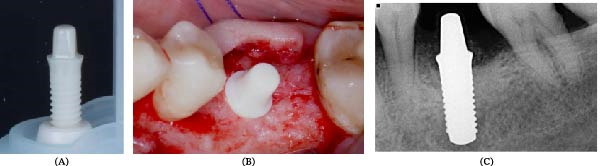
(A) Conventional zirconia implant before placement. (B) An intraoperative photograph showing implant insertion into the prepared osteotomy site; measurement markers on the periodontal probe reflect spatial orientation. (C) Postoperative periapical radiograph acquired using a standardized long‐cone paralleling technique, with millimeter calibration for evaluation of implant seating and crestal bone levels.

Participants received a standard zirconia dental implant, a Straumann PURETM Ceramic Implant with a 3.3 mm diameter and an 8 mm intrabony length, and a 1.8 mm transmucosal height, placed according to established surgical protocols (Figure 2A,B). The same postoperative instructions applied to the experimental group were followed to ensure consistency in oral hygiene practices and healing conditions (Figure 2C).

### 2.5. Follow‐Up

Both groups were scheduled for follow‐up visits at 1 and 3 months postimplantation. Clinical assessments at each visit included evaluation of oral hygiene status through PI and BOP measurements. The surgical site was examined for signs of inflammation, infection, swelling, or implant mobility, the latter of which indicates potential implant failure. CBCT imaging was repeated to monitor changes in peri‐implant bone levels and to detect any signs of bone resorption. The data were analyzed to compare the clinical performance of RAIs with that of CZIs during short‐term healing.

### 2.6. Statistical Analysis

Statistical analyses were conducted using IBM SPSS Statistics (Version 23; Armonk, NY: IBM Corp.). Descriptive statistics were calculated, including means and standard deviations for continuous variables, and frequencies and percentages for categorical variables. Data normality was confirmed using the Shapiro–Wilk test (*p* > 0.05). Between‐group comparisons for continuous variables were performed using independent‐samples *t*‐tests, with corresponding 95% confidence intervals (CIs) and effect sizes (Cohen’s d) reported. Categorical variables were analyzed using chi‐square tests, with Cramer’s *V* reported as the effect size. All tests were two‐tailed, and the significance level was set at *p*  < 0.05.

## 3. Results

### 3.1. Participant Characteristics

Thirty participants were recruited, with 15 receiving CZIs and 15 receiving RAIs. There was no significant difference in age (40.5 ± 8.5 years in the CZI group vs. 36.5 ± 11.1 years in the RAI group; *p* = 0.30; 95% CI: −3.8 to 11.8; Cohen’s *d* = 0.41) or gender distribution (*p* = 0.70; Cramer’s *V* = 0.10) between groups (Table 1).

**Table 1:** Demographic characteristics of the sample.

Group	Age (Mean ± SD)	*p*‐Value	Gender		*p*‐Value
			Male *N* (%)	Female *N* (%)	
CZIs (*N* = 15)	40.5 ± 8.5	0.3	4 (26.7)	11 (73.3%)	0.7
RAIs (*N* = 15)	36.5 ± 11.1		5 (33.3)	10 (66.7)	

### 3.2. Preoperative Clinical and Radiographic Measurement

Preoperative assessment demonstrated a statistically significant difference in baseline bone height between groups (CZIs: 7.7 ± 0.5 mm vs. RAIs: 7.3 ± 0.5 mm; *p* = 0.03; 95% CI: 0.03–0.77; *d* = 0.80). However, there were no significant differences in gingival classification (*p* = 0.07; Cramer’s *V* = 0.32), preoperative PI (*p* = 0.40; *d* = 0.32), or BOP (*p* = 0.06; *d* = 0.69) (Table 2).

**Table 2:** Preoperative clinical and radiographic measurement *N* = 15.

Variable	CZIs (*N* = 15)	RAIs (*N* = 15)	*p*‐Value
Gingival classification			
Healthy *N* (%)	5 (33.3%)	10 (66.7%)	0.07
Gingivitis *N* (%)	10 (66.7%)	5 (33.3%)	
Plaque index (%) (Mean ± SD)	17.5 ± 7.0	19.3 ± 4.0	0.40
Bleeding on probing (%) (Mean ± SD)	17.0 ± 9.6	11.3 ± 5.1	0.06
Bone height (%) (Mean ± SD)	7.7 ± 0.5	7.3 ± 0.5	0.03 ^∗^

^∗^ *p*  < 0.05.

### 3.3. Oral Hygiene and Bone Height Assessments

At the 1‐month postoperative follow‐up, PI values remained comparable between groups (*p* = 0.30; 95% CI: −8.7 to 2.7; *d* = 0.54). BOP was lower in the RAI group and approached statistical significance (*p* = 0.06; mean difference 4.1%; 95% CI:−0.2 to 8.4; *d* = 0.82). A significant difference in bone height was observed, with a mean difference of –0.40 mm (*p* = 0.02; 95% CI: –0.73 to –0.07; *d* = 0.72) (Table 3).

**Table 3:** Postoperative oral hygiene and bone height assessments *N* = 15.

	After 1 month			After 3 months		
Variable	CZIs	RAIs	*p*‐Value	CZIs	RAIs	*p*‐Value
Plaque index (%), (Mean ± SD)	16.6 ± 6.6	19.6 ± 4.4	0.30	18.9 ± 16.2	15.2 ± 3.9	0.40
Bleeding on probing (%), (Mean ± SD)	14.3 ± 6.5	10.2 ± 3.4	0.06	15.0 ± 9.0	13.1 ± 5.3	0.50
Bone height (mm), (Mean ± SD)	7.3 ± 0.5	7.7 ± 0.6	0.02	7.3 ± 0.5	7.5 ± 0.6	0.02 ^∗^

^∗^ *p* < 0.05.

**Figure 3:**
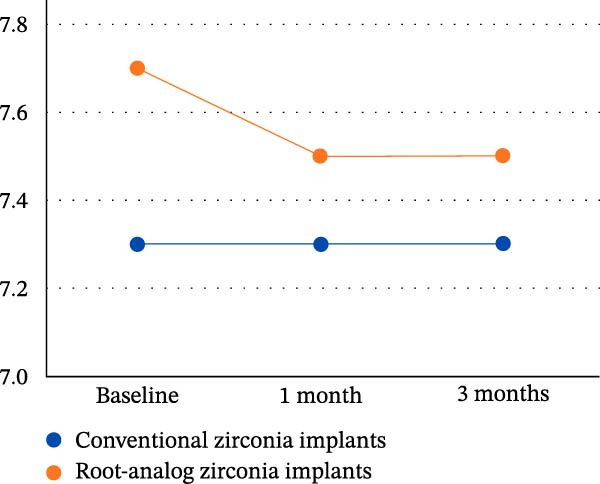
Chart showing mean height of the bone crest (mm) assessed by CBCT at preoperative, 1 month, and 3 months postsurgery for zirconia and root‐analog implants.

At 3 months, PI values were not significantly different between groups (*p* = 0.40; 95% CI: –5.3 to 12.7; *d* = 0.29). Similarly, BOP was comparable (*p* = 0.50; mean difference 1.9%; 95% CI: –4.2 to 8.0; *d* = 0.24). Bone height measurements continued to differ significantly (*p* = 0.02; 95% CI: –0.37 to –0.03; *d* = 0.36) (Figure 3).

### 3.4. Complications

**Figure 4:**
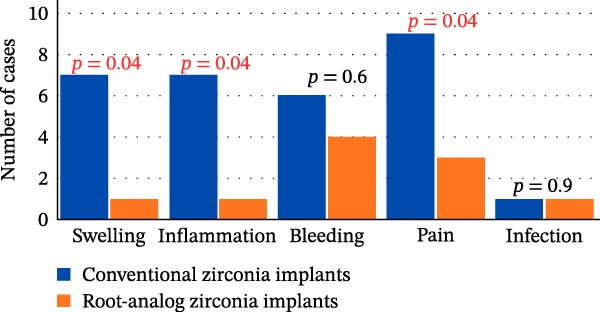
Summary of postoperative complications at the 1‐month follow‐up. Data represent clinically recorded outcomes (swelling, pain, inflammation, bleeding, and infection) based on standardized clinical examination criteria; values plotted on a uniform scale for cross‐group comparison.

**Figure 5:**
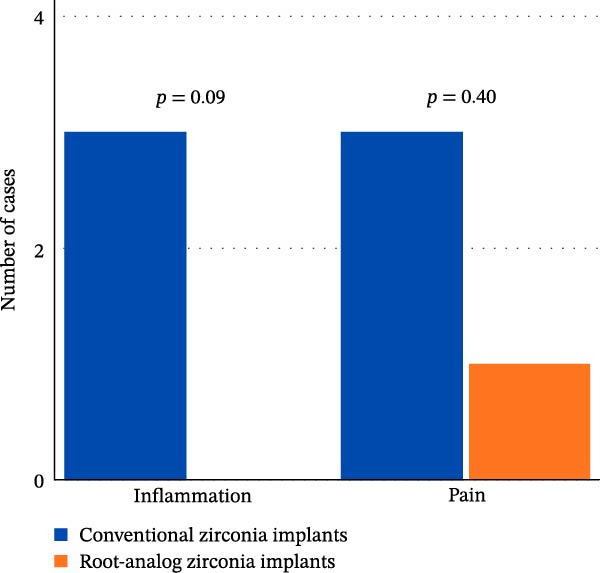
Summary of postoperative complications at the 3‐month follow‐up.

Significantly higher rates of swelling, pain, and inflammation were observed in the CZI group (*p* = 0.04; Cramer’s *V* = 0.37–0.40), while bleeding (*p* = 0.60; *V* < 0.10) and infection (*p* = 0.90; *V* < 0.10) did not differ between groups (Figure 4). At the 3‐month review, no significant differences were observed in inflammation (*p* = 0.09; *V* = 0.20) or pain (*p* = 0.40; *V* = 0.25) (Figure 5).

### 3.5. Discussion

The present study was conducted to evaluate and compare soft tissue response and bone‐preservation patterns between CZIs and RAIs, revealing a statistically significant difference in bone crest height between the two groups at 1 and 3 months. However, there are no statistically significant differences in postoperative PI or BOP between the groups. This outcome can be attributed to the anatomical congruence of RAIs, which are designed to replicate the natural root morphology and fit precisely within the extraction socket. Such a design may reduce micromovements and distribute occlusal forces more evenly, thereby minimizing stress‐induced bone resorption. This interpretation is supported by finite element analysis studies examining stress distributions around RAIs. One study demonstrated that sagittal root position significantly influences stress concentration, with Class II positions showing the most favorable stress distribution under both vertical and oblique loading. Importantly, the von Mises stress values around RAIs were consistently lower than those reported for conventional implants, indicating a biomechanical advantage that may translate into better clinical outcomes [36].

A patient‐specific study evaluating five custom RAI designs demonstrated that the “Plug” design had the lowest von Mises stress. In contrast, the “Fins” and “Bulbs” designs showed reduced contact separation, suggesting improved primary stability. These findings highlight the potential of targeted press‐fit geometries to enhance stress distribution and reduce bone strain, thereby contributing to the improved bone height preservation observed in our clinical data [20]. Similar plaque accumulation and BOP suggest comparable peri‐implant soft tissue health, which is critical for implant longevity. According to existing evidence on the influence of soft tissue condition and plaque accumulation around dental implants on peri‐implantitis development, soft tissue response often correlates more with oral hygiene than with implant design [37].

At 1‐month follow‐up, there are statistically significant differences in swelling, pain, and inflammation between the two groups, with the CZI group experiencing these complications more frequently. A higher incidence of these complications in CZIs may relate to differences in implant surface or design that affect the initial tissue response. This observation is consistent with findings from a recent systematic review of clinical trials and case reports on customized RAIs. The review highlighted that RAIs with specific design features, such as macroretentions, were associated with successful clinical outcomes and showed no signs of infection, bleeding, or peri‐implant inflammation during follow‐up [13]. Also, supporting evidence from a recent systematic review evaluating long‐term outcomes of RAIs demonstrated high survival rates (97.2%) and minimal marginal bone loss (mean 1.1 mm) over 5 years [1]. The review also reported low probing depths and a reduced incidence of biological complications, suggesting that zirconia RAIs may contribute to improved peri‐implant tissue health and reduced inflammatory response. These results suggest that RAIs may offer biological advantages by preserving surrounding tissues and reducing the need for secondary surgical interventions, thereby enhancing patient comfort and satisfaction.

The current study shows that there are no statistically significant differences between the two groups in inflammation and pain after 3 months. The reduction in early complications suggests favorable tissue integration over time, with no long‐term difference between groups. This aligns with findings from a narrative review that observed that peri‐implant soft tissue responses stabilize after the initial healing period, regardless of implant design [38].

The present study found a statistically significant difference in bone height between the zirconia RAI group and the CZI group preoperatively. The significant difference suggests initial anatomical variability, which could influence postoperative healing and bone preservation. This underscores the importance of baseline assessment, as preoperative bone height can impact implant stability and success. Moreover, the comparative effectiveness of RAIs in maintaining marginal bone levels supports the notion, as proposed by a previous study, that marginal bone loss within the first 6 months is a critical indicator of long‐term implant success [39]. While their study established a threshold of 0.5 mm MBL as a prognostic criterion, our results suggest that RAIs may help mitigate early bone loss, thereby potentially improving long‐term outcomes [39].

According to the bone crest height, the constant bone crest height over time indicates that both implant types effectively maintain marginal bone levels, which is vital for implant longevity. The slight initial advantage for RAIs might be due to their anatomical design, which provides better load distribution. This observation agrees with a review finding that RAIs tend to promote less early marginal bone loss due to their close mimicry of the natural root morphology, potentially reducing biomechanical stress concentrations [40].

Regarding the PI, the absence of significant differences in plaque accumulation suggests that both implant types are equally susceptible to plaque retention, emphasizing the importance of oral hygiene rather than implant design alone. This is in line with a study that showed plaque accumulation is primarily influenced by patient oral hygiene practices, and implant surface characteristics play a secondary role [10]. The current study indicates that zirconia RAIs may offer advantages in early soft tissue response and crestal bone preservation, likely due to their anatomical design, which aligns with recent literature emphasizing the benefits of root‐analog approaches [10, 41].

### 3.6. Limitations of the Study

This study has several limitations. The small sample size reduces statistical power and may affect reliability, limiting generalizability to broader populations. Although this study provides valuable insights into early postoperative healing around both RAIs and CZIs, the 3‐month follow‐up period represents a short‐term evaluation. Implant success, marginal bone stability, and biological complications are typically assessed over longer intervals, often ≥6–12 months. Therefore, the present findings should be interpreted within the context of early healing only, and no long‐term conclusions can be drawn. Extended follow‐up studies are warranted to determine the medium‐ and long‐term performance of RAIs relative to established zirconia implant systems. The focus on single‐rooted teeth simplifies anatomical variability but restricts applicability to complex cases such as multirooted teeth or compromised bone conditions. The use of customized RAIs introduces procedural variability, as factors including digital design precision, milling accuracy, surface treatment, and insertion technique may influence outcomes and reproducibility. Finally, although zirconia offers favorable biological and esthetic properties, its long‐term mechanical performance, particularly its fracture resistance under functional loading, remains uncertain and warrants further investigation.

Also, a statistically significant difference in baseline preoperative bone height was observed between the two groups. This imbalance may have influenced postoperative radiographic measurements and should be considered when interpreting the findings. Although both groups followed standardized surgical and follow‐up protocols, variations in initial bone levels remain a potential confounding factor that could partially account for postoperative outcomes.

The study did not employ random allocation to groups, which may introduce selection bias and limit internal validity. Primary implant stability was also not measured at placement, preventing evaluation of its influence on early healing responses.

### 3.7. Future Research Directions

Future studies should include larger‐scale, multicenter randomized controlled trials with extended follow‐up periods to assess long‐term outcomes. Investigating patient‐reported measures, cost‐effectiveness, and the influence of various surface treatments on implant performance would provide a more comprehensive understanding. Such studies could further refine clinical protocols and support evidence‐based decision‐making in the use of zirconia RAIs.

## 4. Conclusions

RAIs demonstrated favorable short‐term healing and reduced early postoperative discomfort compared with CZI. These findings highlight potential biological advantages of anatomically customized implant designs; however, the short follow‐up period and nonrandomized group allocation limit broader interpretation. Longer‐term comparative studies are needed to evaluate the stability and clinical predictability of RAIs.

## Author Contributions

Conceptualization, investigation: Ammar Almarghlani, Amirah Aldosari, and Abdullah Alfarran. Methodology: Ammar Almarghlani, Amirah Aldosari, Abdullah Alfarran, and Ali Alghamdi. Software: Rayan Sharka, Hassan Abed, and Badr Othman. Validation, supervision: Ammar Almarghlani. Formal analysis: Amirah Aldosari, Abdullah Alfarran, and Rayan Sharka. Resources: Ammar Almarghlani, Ali Alghamdi, and Badr Othman. Data curation, visualization: Rayan Sharka and Hassan Abed. Writing – original draft: Amirah Aldosari and Abdullah Alfarran. Writing – review and editing: Ammar Almarghlani, Rayan Sharka, Hassan Abed, Badr Othman, and Ali Alghamdi. Project administration: Ammar Almarghlani, Hassan Abed, and Badr Othman.

## Funding

The authors extend their appreciation to Umm Al‐Qura University, Saudi Arabia, for funding this research work through Grant 26UQU4350291GSSR03.

## Ethics Statement

All the procedures of this research protocol adhered to the Declaration of Helsinki and were approved by the Ethics Committee of King Abdulaziz University (Approval Number 172‐11‐24).

## Conflicts of Interest

The authors declare no conflicts of interest.

## Supporting Information

Additional supporting information can be found online in the Supporting Information section.

## Supporting information


**Supporting Information** The STROBE checklist used to guide the reporting of this observational study is provided as a Supporting Information file.

## Data Availability

The data that support the findings of this study are available from the corresponding author upon reasonable request.
